# Crystal structure of *trans*-di­aqua­bis­(1*H*-pyrazole-3-carboxyl­ato-κ^2^ *N*,*O*)copper(II) dihydrate

**DOI:** 10.1107/S2056989015021593

**Published:** 2015-11-21

**Authors:** Santiago Reinoso, Beñat Artetxe, Oscar Castillo, Antonio Luque, Juan M. Gutiérrez-Zorrilla

**Affiliations:** a Departamento de Química Inorgánica, Facultad de Ciencia y Tecnología, Universidad de País Vasco UPV/EHU, PO Box 644, E-48080 Bilbao, Spain

**Keywords:** crystal structure, copper(II) complex, *trans* configuration, 1*H*-pyrazole-3-carboxyl­ate

## Abstract

In the title compound, [Cu(C_4_H_3_N_2_O_2_)_2_(H_2_O)_2_]·2H_2_O, the Cu^II^ ion is located on an inversion centre and exhibits an axially elongated octa­hedral coordination geometry. The equatorial plane is formed by two *N*,*O*-bidentate 1*H*-pyrazole-3-carboxyl­ate ligands in a *trans* configuration. The axial positions are occupied by two water mol­ecules. The mononuclear complex mol­ecules are arranged in layers parallel to the *ab* plane. Each complex mol­ecule is linked to four adjacent species through inter­molecular O—H⋯O and N—H⋯O hydrogen bonds that are established between the coordinating water mol­ecules and carboxyl­ate O atoms or protonated N atoms of the organic ligands. These layers are further connected into a three-dimensional network by additional hydrogen bonds involving solvent water mol­ecules and non-coordinating carboxyl­ate O atoms.

## Related literature   

For mononuclear cobalt(II), nickel(II) and zinc complexes of the 1*H*-pyrazole-3-carboxyl­ate ligand, see: Artetxe *et al.* (2015 ▸); López-Viseras *et al.* (2014 ▸).
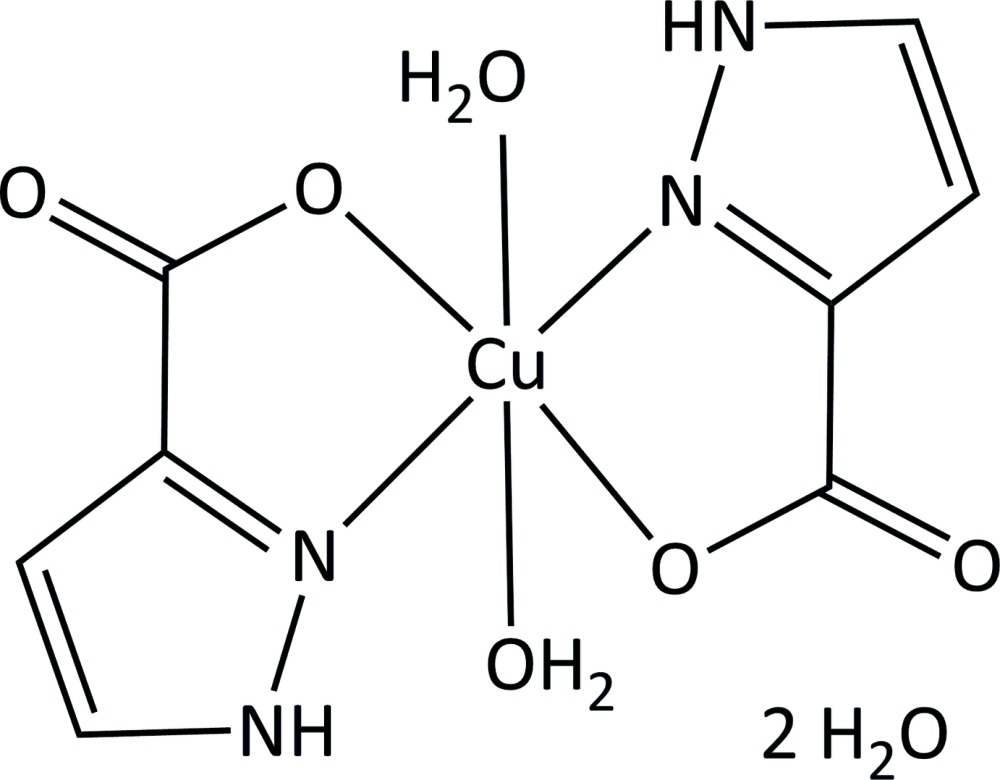


## Experimental   

### Crystal data  

[Cu(C_4_H_3_N_2_O_2_)_2_(H_2_O)_2_]·2H_2_O
*M* *_r_* = 357.77
Monoclinic, 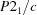

*a* = 6.4780 (4) Å
*b* = 21.5757 (10) Å
*c* = 4.8937 (3) Å
β = 105.856 (7)°
*V* = 657.96 (6) Å^3^
*Z* = 2
Cu *K*α radiation
μ = 2.83 mm^−1^
*T* = 100 K
0.09 × 0.04 × 0.02 mm

### Data collection  

Agilent SuperNova Single source at offset diffractometer
Absorption correction: multi-scan (*CrysAlis PRO*; Agilent, 2011 ▸) *T*_min_ = 0.817, *T*_max_ = 1
4452 measured reflections
1216 independent reflections
1089 reflections with *I* > 2σ(*I*)
*R*_int_ = 0.031

### Refinement  

*R*[*F* ^2^ > 2σ(*F* ^2^)] = 0.028
*wR*(*F* ^2^) = 0.074
*S* = 1.09
1216 reflections
109 parameters
4 restraints
H atoms treated by a mixture of independent and constrained refinement
Δρ_max_ = 0.36 e Å^−3^
Δρ_min_ = −0.35 e Å^−3^

### 

Data collection: *CrysAlis PRO* (Agilent, 2011 ▸); cell refinement: *CrysAlis PRO*; data reduction: *CrysAlis PRO*; program(s) used to solve structure: *OLEX2* (Dolomanov *et al.*, 2009 ▸); program(s) used to refine structure: *SHELXL97* (Sheldrick, 2008 ▸); molecular graphics: *ORTEP-3 for Windows* (Farrugia, 2012 ▸); software used to prepare material for publication: *WinGX* (Farrugia, 2012 ▸) and *PLATON* (Spek, 2009 ▸).

## Supplementary Material

Crystal structure: contains datablock(s) I, global. DOI: 10.1107/S2056989015021593/im2473sup1.cif

Structure factors: contains datablock(s) global, a2013072_cupyc. DOI: 10.1107/S2056989015021593/im2473Isup2.hkl

**Fig. 1.**
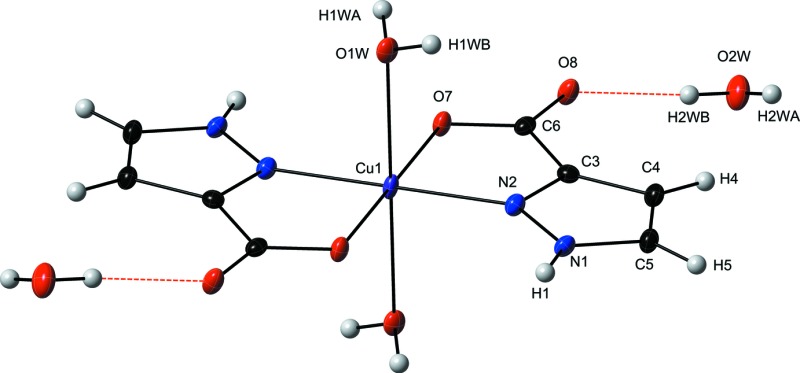
Molecular structure of [Cu(C4H3N2O2)2(H2O)2] ·2H2O showing the atom labelling for the asymmetric unit and 50% probability displacement ellipsoids.

**Fig. 2.**
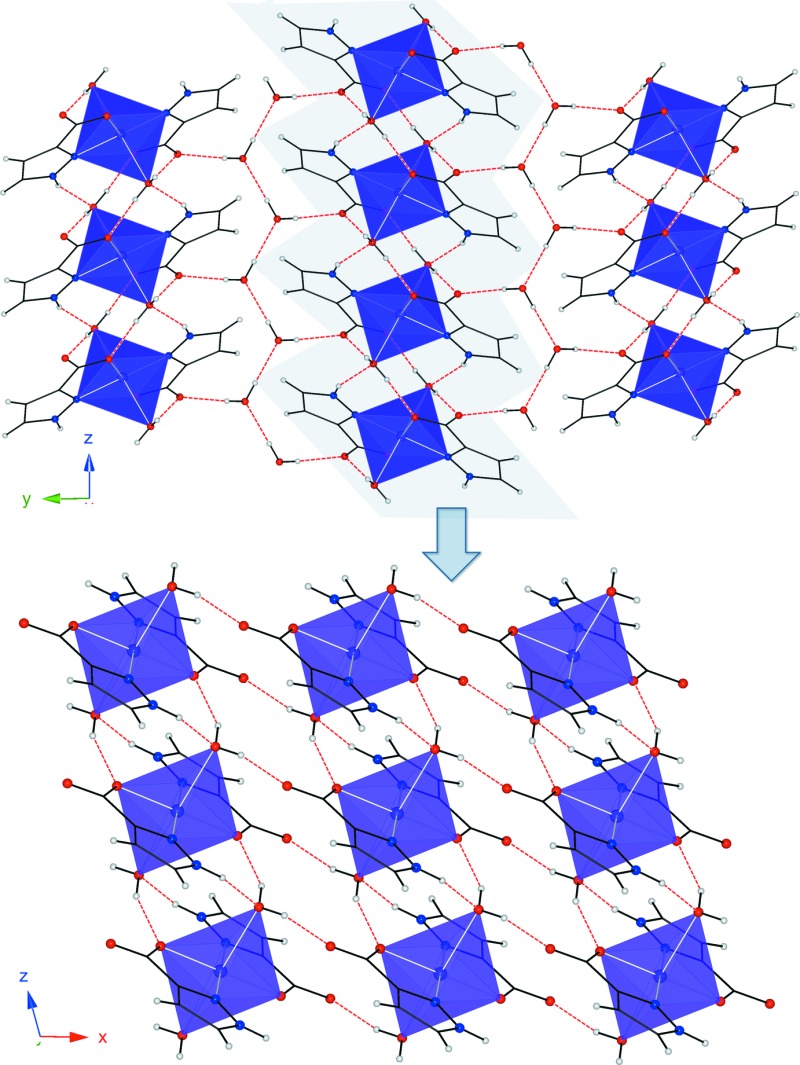
View of the crystal packing along the crystallographic a axis (above). Projection of a layer of [Cu(C4H3N2O2)2(H2O)2] complexes along the [010] direction (below). Cu(II) centres are represented as translucent octahedra and the O—H···O and N—H···O hydrogen bonds are depicted as dashed red lines.

4 3 2 2 2 2 2 2 . DOI: 10.1107/S2056989015021593/im2473fig1.tif
Mol­ecular structure of [Cu(C_4_H_3_N_2_O_2_)_2_(H_2_O)_2_] ·2H_2_O showing the atom labelling for the asymmetric unit and 50% probability displacement ellipsoids.

a 4 3 2 2 2 2 2 . DOI: 10.1107/S2056989015021593/im2473fig2.tif
View of the crystal packing along the crystallographic *a* axis (above). Projection of a layer of [Cu(C_4_H_3_N_2_O_2_)_2_(H_2_O)_2_] complexes along the [010] direction (below). Cu(II) centres are represented as translucent octa­hedra and the O—H⋯O and N—H⋯O hydrogen bonds are depicted as dashed red lines.

CCDC reference: 1437048

Additional supporting information:  crystallographic information; 3D view; checkCIF report

## Figures and Tables

**Table:** 

Cu1—N2	1.9808 (16)
Cu1—O7	1.9910 (14)
Cu1—O1*W*	2.4501 (15)

**Table:** 

N2—Cu1—O7	81.30 (6)
N2—Cu1—O1*W*	92.08 (6)
O7—Cu1—O1*W*	89.43 (5)

**Table 2:** Hydrogen-bond geometry (Å, °)

*D*—H⋯*A*	*D*—H	H⋯*A*	*D*⋯*A*	*D*—H⋯*A*
N1—H1⋯O1*W* ^i^	0.88	1.93	2.710 (2)	147
O1*W*—H1*WA*⋯O8^ii^	0.83 (2)	1.86 (2)	2.667 (2)	163 (3)
O1*W*—H1*WB*⋯O7^iii^	0.82 (2)	1.96 (2)	2.709 (2)	153 (3)
O2*W*—H2*WA*⋯O2*W* ^iv^	0.83 (2)	1.95 (2)	2.7792 (15)	178 (3)
O2*W*—H2*WB*⋯O8	0.81 (2)	2.04 (2)	2.854 (2)	175 (3)

Symmetry codes: (i) 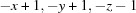; (ii) 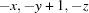; (iii) 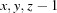; (iv) 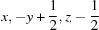.

## supplementary crystallographic information

### S1. Structural commentary

The title compound, [Cu(C_4_H_3_N_2_O_2_)_2_(H_2_O)_2_] ·2H_2_O crystallizes in the monoclinic crystal system, space group *P*2_1_/*c*. The equatorial Cu—O and Cu—N distances (Table 1) are similar to those observed for the corresponding Co(II), Ni(II) and Zn(II) analogues (Artetxe *et al.*, 2015; López-Viseras *et al.*, 2014). However, the axial bond lenghts are much longer due to the Jahn-Teller effect operating in Cu(II) centres. The mononuclear complexes arrange in layers parallel to the *ab* plane through inter­molecular O—H···O and N—H···O hydrogen bonds that are established between the coordinated water molecules (O1W) and carboxyl­ate O atoms (O7, O8) or protonated N atoms (N2) of the organic ligands. These layers are further connected into a three-dimensional network by additional hydrogen bonds involving solvent water molecules (O2W) and non-coordinating carboxyl­ate O atoms (O8). Table 2 summarizes the geometrical parameters of these O—H···O and N—H···O hydrogen bonding inter­actions.

### S2. Synthesis and crystallization

To a solution of CuCl_2_ · 2 H_2_O (51 mg, 0.3 mmol) in hot water (15 ml) 1*H*-pyrazole-3-carb­oxy­lic acid (74 mg, 0.6 mmol) dissolved in hot water (10 ml) was added dropwise. After stirring for 30 min at 90 °C, the final solution was left undisturbed and prismatic blue crystals suitable for X-ray diffraction were obtained upon cooling to room temperature (Yield: 68 mg, 63%).

### S3. Refinement

Crystal data, data collection and structure refinement details are summarized in Table 1. All atoms except H were refined anisotropically. H atoms of the water molecules were located in a Fourier difference map and refined isotropically with O—H bond lenghts restrained to 0.84 (2) and with *U*_iso_(H) = 1.5*U*_eq_(O). All pyrazole H atoms were positioned geometrically and refined using a riding model with C—H = 0.95 Å, N—H = 0.88 Å and *U*_iso_(H) = 1.2*U*_eq_(C,*N*).

### Crystal data

**Table:**

[Cu(C_4_H_3_N_2_O_2_)_2_(H_2_O)_2_]·2H_2_O	*F*(000) = 366
*M**_r_* = 357.77	*D*_x_ = 1.806 Mg m^−^^3^
Monoclinic, *P*2_1_/*c*	Cu *K*α radiation, λ = 1.54184 Å
Hall symbol: -P 2ybc	Cell parameters from 1956 reflections
*a* = 6.4780 (4) Å	θ = 4.1–73.7°
*b* = 21.5757 (10) Å	µ = 2.83 mm^−^^1^
*c* = 4.8937 (3) Å	*T* = 100 K
β = 105.856 (7)°	Prism, blue
*V* = 657.96 (6) Å^3^	0.09 × 0.04 × 0.02 mm
*Z* = 2	

### Data collection

**Table:**

Agilent SuperNova Single source at offset diffractometer	1216 independent reflections
Radiation source: SuperNova (Cu) X-ray Source	1089 reflections with *I* > 2σ(*I*)
Mirror monochromator	*R*_int_ = 0.031
Detector resolution: 5.2012 pixels mm^-1^	θ_max_ = 69°, θ_min_ = 4.1°
ω scans	*h* = −6→7
Absorption correction: multi-scan (*CrysAlis PRO*; Agilent, 2011)	*k* = −23→26
*T*_min_ = 0.817, *T*_max_ = 1	*l* = −5→5
4452 measured reflections	

### Refinement

**Table:**

Refinement on *F*^2^	Primary atom site location: structure-invariant direct methods
Least-squares matrix: full	Secondary atom site location: difference Fourier map
*R*[*F*^2^ > 2σ(*F*^2^)] = 0.028	Hydrogen site location: inferred from neighbouring sites
*wR*(*F*^2^) = 0.074	H atoms treated by a mixture of independent and constrained refinement
*S* = 1.09	*w* = 1/[σ^2^(*F*_o_^2^) + (0.0406*P*)^2^ + 0.2735*P*] where *P* = (*F*_o_^2^ + 2*F*_c_^2^)/3
1216 reflections	(Δ/σ)_max_ < 0.001
109 parameters	Δρ_max_ = 0.36 e Å^−^^3^
4 restraints	Δρ_min_ = −0.35 e Å^−^^3^

### Special details

**Table:**

Experimental. IR (KBr pellets, cm^-1^): 3487(*s*), 3340(*s*), 3140(*s*), 3075(*s*), 2854(*s*), 2795(*s*), 1695(*s*), 1501(*m*), 1451(w), 1358(*s*), 1263(w), 1132(w), 1069(w), 1015(w), 943(*m*), 899(*m*), 839(*m*), 785(*m*), 648(*m*), 615(w), 500(w).TGA/DTA (synthetic air, 5°C min^-1^): The initial endothermic dehydration proccess (calcd/found for 4H_2_O: 20.1 /20.2%) is completed at c.a. 85°C and is followed by a thermal stability range for the anydrous phase that extends up to c.a. 210°C. The highly exothermic ligand combustion results in the final residue at 370°C (calcd/found for CuO: 22.1/21.8%).CHN (%m, calcd/found): C (26.8/27.2), H (3.9/3.9), N(15.7/15.5).
Geometry. All e.s.d.'s (except the e.s.d. in the dihedral angle between two l.s. planes) are estimated using the full covariance matrix. The cell e.s.d.'s are taken into account individually in the estimation of e.s.d.'s in distances, angles and torsion angles; correlations between e.s.d.'s in cell parameters are only used when they are defined by crystal symmetry. An approximate (isotropic) treatment of cell e.s.d.'s is used for estimating e.s.d.'s involving l.s. planes.
Refinement. Refinement of *F*^2^ against ALL reflections. The weighted *R*-factor *wR* and goodness of fit *S* are based on *F*^2^, conventional *R*-factors *R* are based on *F*, with *F* set to zero for negative *F*^2^. The threshold expression of *F*^2^ > σ(*F*^2^) is used only for calculating *R*-factors(gt) *etc*. and is not relevant to the choice of reflections for refinement. *R*-factors based on *F*^2^ are statistically about twice as large as those based on *F*, and *R*- factors based on ALL data will be even larger.

### Fractional atomic coordinates and isotropic or equivalent isotropic displacement parameters (Å^2^)

**Table:**

	*x*	*y*	*z*	*U*_iso_*/*U*_eq_	
Cu1	0.5	0.5	0	0.01043 (15)	
N1	0.5203 (3)	0.37840 (7)	−0.3376 (4)	0.0113 (4)	
H1	0.6239	0.3877	−0.4153	0.014*	
N2	0.4416 (3)	0.41696 (7)	−0.1769 (4)	0.0111 (4)	
C3	0.2890 (3)	0.38581 (9)	−0.0975 (4)	0.0105 (4)	
C4	0.2691 (3)	0.32597 (9)	−0.2127 (4)	0.0124 (4)	
H4	0.1732	0.2942	−0.1909	0.015*	
C5	0.4182 (3)	0.32329 (9)	−0.3637 (4)	0.0137 (4)	
H5	0.4452	0.2886	−0.4687	0.016*	
C6	0.1815 (3)	0.42089 (9)	0.0858 (4)	0.0107 (4)	
O7	0.2562 (2)	0.47569 (6)	0.1520 (3)	0.0118 (3)	
O8	0.0330 (2)	0.39735 (6)	0.1641 (3)	0.0144 (3)	
O1W	0.2455 (2)	0.54965 (6)	−0.4047 (3)	0.0134 (3)	
O2W	−0.1631 (3)	0.28054 (7)	0.2052 (4)	0.0219 (4)	
H1WA	0.140 (4)	0.5615 (13)	−0.353 (6)	0.033*	
H1WB	0.208 (5)	0.5252 (12)	−0.536 (5)	0.033*	
H2WA	−0.167 (5)	0.2621 (13)	0.054 (5)	0.033*	
H2WB	−0.102 (4)	0.3132 (10)	0.201 (6)	0.033*	

### Atomic displacement parameters (Å^2^)

**Table:**

	*U*^11^	*U*^22^	*U*^33^	*U*^12^	*U*^13^	*U*^23^
Cu1	0.0144 (2)	0.0066 (2)	0.0126 (2)	−0.00226 (14)	0.00748 (17)	−0.00236 (15)
N1	0.0134 (8)	0.0095 (8)	0.0118 (8)	0.0020 (6)	0.0049 (7)	−0.0013 (6)
N2	0.0139 (8)	0.0095 (8)	0.0105 (8)	0.0006 (6)	0.0046 (7)	−0.0006 (6)
C3	0.0101 (9)	0.0116 (9)	0.0091 (9)	−0.0005 (7)	0.0016 (7)	0.0019 (7)
C4	0.0147 (10)	0.0089 (9)	0.0136 (10)	−0.0006 (8)	0.0039 (8)	0.0008 (7)
C5	0.0162 (10)	0.0094 (9)	0.0149 (10)	0.0008 (7)	0.0035 (8)	−0.0019 (7)
C6	0.0124 (9)	0.0098 (9)	0.0093 (10)	0.0026 (7)	0.0021 (8)	0.0013 (7)
O7	0.0153 (7)	0.0087 (6)	0.0132 (7)	−0.0015 (5)	0.0071 (5)	−0.0020 (5)
O8	0.0159 (7)	0.0106 (7)	0.0198 (8)	−0.0013 (5)	0.0103 (6)	0.0000 (6)
O1W	0.0153 (7)	0.0130 (7)	0.0140 (7)	−0.0010 (6)	0.0076 (6)	−0.0037 (6)
O2W	0.0313 (9)	0.0143 (7)	0.0209 (9)	−0.0057 (7)	0.0084 (7)	0.0011 (7)

### Geometric parameters (Å, º)

**Table:**

Cu1—N2	1.9808 (16)	C3—C6	1.484 (3)
Cu1—N2^i^	1.9808 (16)	C4—C5	1.369 (3)
Cu1—O7^i^	1.9910 (14)	C4—H4	0.95
Cu1—O7	1.9910 (14)	C5—H5	0.95
Cu1—O1W	2.4501 (15)	C6—O8	1.238 (3)
Cu1—O1W^i^	2.4501 (15)	C6—O7	1.285 (2)
N1—N2	1.338 (2)	O1W—H1WA	0.833 (18)
N1—C5	1.350 (3)	O1W—H1WB	0.817 (18)
N1—H1	0.88	O2W—H2WA	0.834 (18)
N2—C3	1.338 (3)	O2W—H2WB	0.813 (17)
C3—C4	1.401 (3)		
			
N2—Cu1—N2^i^	180.00 (4)	N1—N2—Cu1	139.59 (13)
N2—Cu1—O7^i^	98.70 (6)	N2—C3—C4	109.90 (18)
N2^i^—Cu1—O7^i^	81.30 (6)	N2—C3—C6	115.16 (17)
N2—Cu1—O7	81.30 (6)	C4—C3—C6	134.95 (18)
N2^i^—Cu1—O7	98.70 (6)	C4—C3—Cu1	150.27 (15)
O7^i^—Cu1—O7	180	C6—C3—Cu1	74.67 (11)
N2—Cu1—O1W	92.08 (6)	C5—C4—C3	104.79 (17)
N2^i^—Cu1—O1W	87.92 (6)	C5—C4—H4	127.6
O7^i^—Cu1—O1W	90.57 (5)	C3—C4—H4	127.6
O7—Cu1—O1W	89.43 (5)	N1—C5—C4	108.16 (18)
N2—Cu1—O1W^i^	87.92 (6)	N1—C5—H5	125.9
N2^i^—Cu1—O1W^i^	92.08 (6)	C4—C5—H5	125.9
O7^i^—Cu1—O1W^i^	89.43 (5)	O8—C6—O7	124.79 (19)
O7—Cu1—O1W^i^	90.57 (5)	O8—C6—C3	120.69 (17)
O1W—Cu1—O1W^i^	180.00 (6)	O7—C6—C3	114.52 (17)
N2—N1—C5	110.36 (17)	O8—C6—Cu1	164.75 (15)
C5—N1—Cu1	134.42 (13)	C3—C6—Cu1	74.54 (11)
N2—N1—H1	124.8	C6—O7—Cu1	115.51 (13)
C5—N1—H1	124.8	Cu1—O1W—H1WA	108 (2)
Cu1—N1—H1	100.7	Cu1—O1W—H1WB	110 (2)
C3—N2—N1	106.79 (16)	H1WA—O1W—H1WB	110 (3)
C3—N2—Cu1	113.30 (14)	H2WA—O2W—H2WB	107 (3)
			
N2^i^—Cu1—N1—N2	180	O7—Cu1—C3—C6	−0.48 (10)
O7^i^—Cu1—N1—N2	−176.5 (2)	O1W—Cu1—C3—C6	−87.49 (11)
O7—Cu1—N1—N2	3.5 (2)	O1W^i^—Cu1—C3—C6	92.51 (11)
O1W—Cu1—N1—N2	−86.4 (2)	N2—C3—C4—C5	0.2 (2)
O1W^i^—Cu1—N1—N2	93.6 (2)	C6—C3—C4—C5	−180.0 (2)
N2—Cu1—N1—C5	−8.9 (2)	Cu1—C3—C4—C5	−6.1 (3)
N2^i^—Cu1—N1—C5	171.1 (2)	N2—N1—C5—C4	−0.5 (2)
O7^i^—Cu1—N1—C5	174.58 (18)	Cu1—N1—C5—C4	3.4 (3)
O7—Cu1—N1—C5	−5.42 (18)	C3—C4—C5—N1	0.2 (2)
O1W—Cu1—N1—C5	−95.26 (18)	N2—C3—C6—O8	177.59 (17)
O1W^i^—Cu1—N1—C5	84.74 (18)	C4—C3—C6—O8	−2.3 (3)
C5—N1—N2—C3	0.6 (2)	Cu1—C3—C6—O8	−179.15 (18)
Cu1—N1—N2—C3	−172.7 (3)	N2—C3—C6—O7	−2.6 (3)
C5—N1—N2—Cu1	173.24 (16)	C4—C3—C6—O7	177.6 (2)
O7^i^—Cu1—N2—C3	175.82 (13)	Cu1—C3—C6—O7	0.68 (14)
O7—Cu1—N2—C3	−4.18 (13)	N2—C3—C6—Cu1	−3.26 (14)
O1W—Cu1—N2—C3	−93.28 (14)	C4—C3—C6—Cu1	176.9 (2)
O1W^i^—Cu1—N2—C3	86.72 (14)	N2—Cu1—C6—O8	179.6 (6)
O7^i^—Cu1—N2—N1	3.5 (2)	N2^i^—Cu1—C6—O8	−0.4 (6)
O7—Cu1—N2—N1	−176.5 (2)	O7^i^—Cu1—C6—O8	176.3 (5)
O1W—Cu1—N2—N1	94.4 (2)	O7—Cu1—C6—O8	−3.7 (5)
O1W^i^—Cu1—N2—N1	−85.6 (2)	O1W—Cu1—C6—O8	−88.9 (5)
N1—N2—C3—C4	−0.4 (2)	O1W^i^—Cu1—C6—O8	91.1 (5)
Cu1—N2—C3—C4	−175.27 (13)	N2—Cu1—C6—O7	−176.66 (15)
N1—N2—C3—C6	179.66 (15)	N2^i^—Cu1—C6—O7	3.34 (15)
Cu1—N2—C3—C6	4.8 (2)	O7^i^—Cu1—C6—O7	180
N1—N2—C3—Cu1	174.8 (2)	O1W—Cu1—C6—O7	−85.20 (13)
N2^i^—Cu1—C3—N2	180	O1W^i^—Cu1—C6—O7	94.80 (13)
O7^i^—Cu1—C3—N2	−5.02 (16)	N2—Cu1—C6—C3	2.38 (10)
O7—Cu1—C3—N2	174.98 (16)	N2^i^—Cu1—C6—C3	−177.62 (10)
O1W—Cu1—C3—N2	87.97 (14)	O7^i^—Cu1—C6—C3	−0.96 (19)
O1W^i^—Cu1—C3—N2	−92.03 (14)	O7—Cu1—C6—C3	179.04 (19)
N2—Cu1—C3—C4	9.0 (2)	O1W—Cu1—C6—C3	93.84 (10)
N2^i^—Cu1—C3—C4	−171.0 (2)	O1W^i^—Cu1—C6—C3	−86.16 (10)
O7^i^—Cu1—C3—C4	4.0 (3)	O8—C6—O7—Cu1	178.80 (15)
O7—Cu1—C3—C4	−176.0 (3)	C3—C6—O7—Cu1	−1.0 (2)
O1W—Cu1—C3—C4	97.0 (3)	N2—Cu1—O7—C6	2.83 (13)
O1W^i^—Cu1—C3—C4	−83.0 (3)	N2^i^—Cu1—O7—C6	−177.17 (13)
N2—Cu1—C3—C6	−175.5 (2)	O1W—Cu1—O7—C6	95.02 (13)
N2^i^—Cu1—C3—C6	4.5 (2)	O1W^i^—Cu1—O7—C6	−84.98 (13)
O7^i^—Cu1—C3—C6	179.52 (10)		

Symmetry code: (i) −*x*+1, −*y*+1, −*z*.

### Hydrogen-bond geometry (Å, º)

**Table:**

*D*—H···*A*	*D*—H	H···*A*	*D*···*A*	*D*—H···*A*
N1—H1···O1*W*^ii^	0.88	1.93	2.710 (2)	147
O1*W*—H1*WA*···O8^iii^	0.83 (2)	1.86 (2)	2.667 (2)	163 (3)
O1*W*—H1*WB*···O7^iv^	0.82 (2)	1.96 (2)	2.709 (2)	153 (3)
O2*W*—H2*WA*···O2*W*^v^	0.83 (2)	1.95 (2)	2.7792 (15)	178 (3)
O2*W*—H2*WB*···O8	0.81 (2)	2.04 (2)	2.854 (2)	175 (3)

Symmetry codes: (ii) −*x*+1, −*y*+1, −*z*−1; (iii) −*x*, −*y*+1, −*z*; (iv) *x*, *y*, *z*−1; (v) *x*, −*y*+1/2, *z*−1/2.
