# Supplementary figures and images for: Crystal structure of trans-di­aqua­bis­(1H-pyrazole-3-carboxyl­ato-κ2 N,O)copper(II) dihydrate
Source: Acta Crystallogr E Crystallogr Commun. 2015 Nov 21;71(Pt 12):m232–3. doi: 10.1107/S2056989015021593 (PMC4719849; doi:10.1107/S2056989015021593)

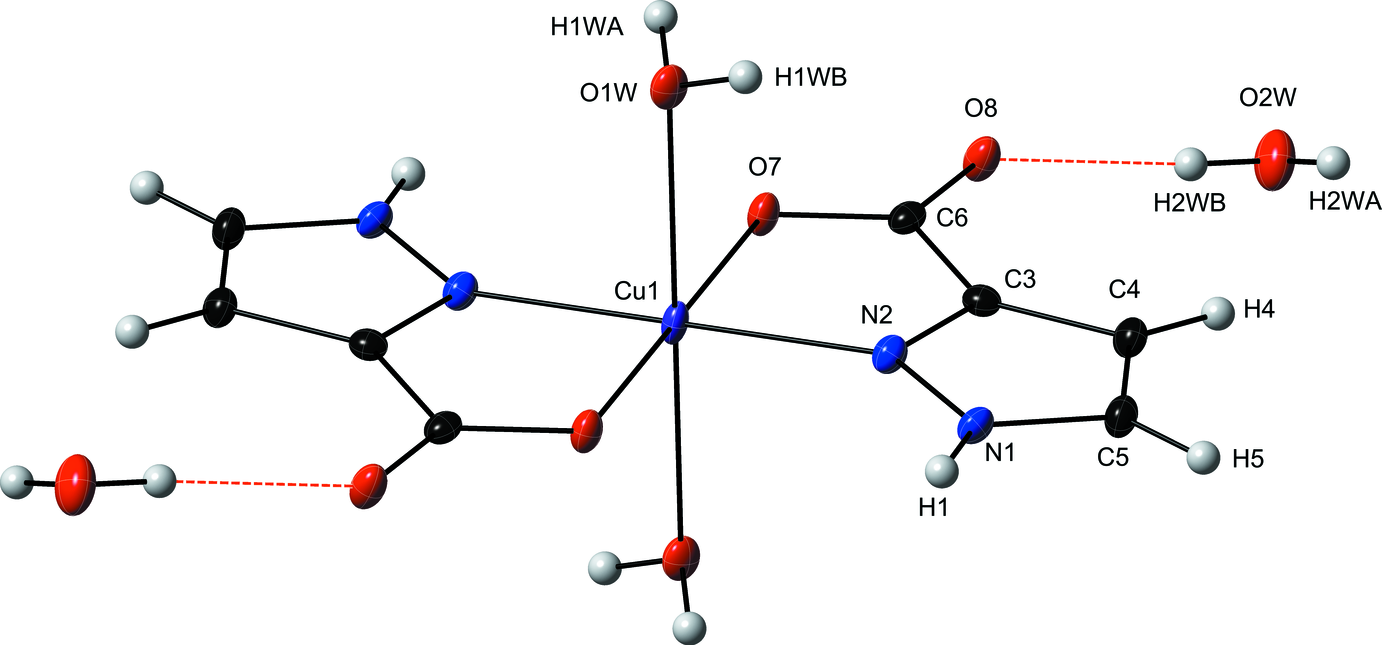

Supplement: Supplementary file 3 [file e-71-0m232-fig1.tif]

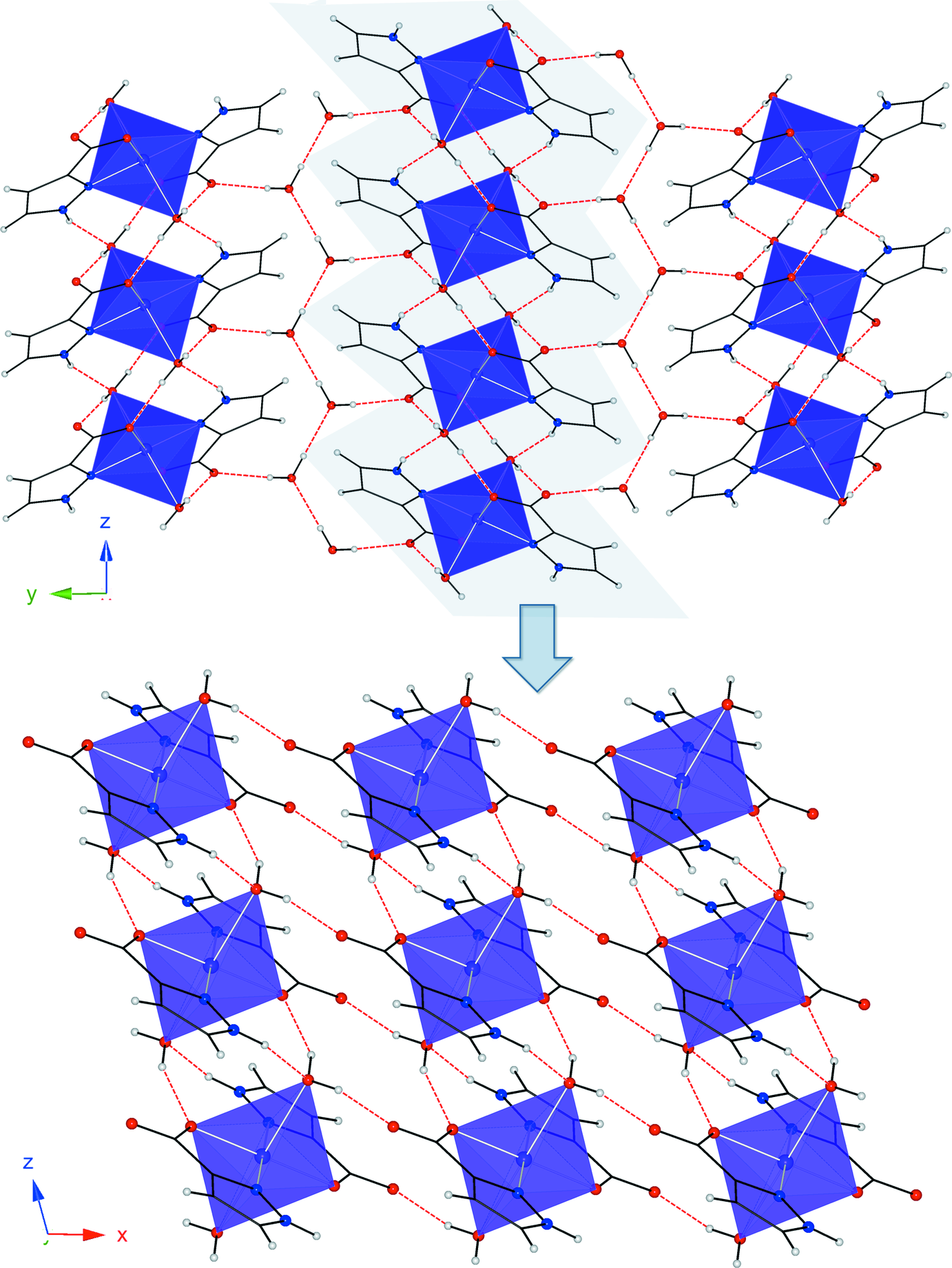

Supplement: Supplementary file 4 [file e-71-0m232-fig2.tif]
